# A New Strategy to Improve Management of Citrus Mal Secco Disease Using Bioformulates Based on *Bacillus* *amyloliquefaciens* Strains

**DOI:** 10.3390/plants11030446

**Published:** 2022-02-06

**Authors:** Dalia Aiello, Giuseppa Rosaria Leonardi, Chiara Di Pietro, Alessandro Vitale, Giancarlo Polizzi

**Affiliations:** Department of Agriculture, Food and Environment (Di3A), University of Catania, Via S. Sofia 100, 95123 Catania, Italy; dalia.aiello@unict.it (D.A.); leonardigiusi@outlook.it (G.R.L.); chiara.dipietro@live.it (C.D.P.); gpolizzi@unict.it (G.P.)

**Keywords:** citrus mal secco disease, *Plenodomus tracheiphilus*, biological control, *Bacillus amyloliquefaciens*

## Abstract

The effectiveness of biological commercial products based on *Bacillus amyloliquefaciens* strains was evaluated through in vitro and in vivo experiments against *Plenodomus tracheiphilus*. The activity of bacterial cells, volatile organic compounds (VOCs), and culture filtrates of bacteria were tested in vitro against different isolates of *P*. *tracheiphilus*. Afterwards, the virulence of these isolates was evaluated on *Citrus volkameriana* plants to select the most virulent isolate to use in the in vivo experiments. To evaluate the effectiveness of products, *C*. *volkameriana* seedlings were pre-treated, twice with biological products and once with standard fungicides, before pathogen inoculation. Moreover, in order to determine the endophytic ability of the bacteria, the population density within the treated citrus stem was determined. Comprehensively, bacterial cells, filtrates, and VOCs were able to significantly reduce the average mycelial diameter of *P*. *tracheiphilus*, with some variability according to pathogen isolate. In planta experiments showed that the biological products on average were less effective than fungicides, although all formulates were able to significantly reduce disease incidence and symptom severity, except *B. amyloliquefaciens* strain D747 (Amylo-X) for symptom severity (SS) 20 days after inoculation. Bacteria were re-isolated from the internal woody tissue of treated plants, showing strong endophytic ability. This work is important as commercial biological products based on *B. amyloliquefaciens* strains could represent a promising and sustainable alternative for the integrated management of mal secco disease.

## 1. Introduction

Citrus represents one of the most important fruit crops in the world. Italy is among the first ten countries in the world, and second in Europe, for orange and lemon production [1]. Italian citrus production is concentrated in the Southern regions, mainly in Sicily and Calabria. Nevertheless, in the last few decades we have seen a consistent reduction in lemon production and area harvested, caused by the mitosporic fungus *Plenodomus tracheiphilus* (Petri) Gruyter, Aveskamp, and Verkley (syn. *Phoma tracheiphila*), the causal agent of mal secco disease [2]. After the first detection in Italy (eastern Sicily) in 1918, the disease rapidly extended to the lemon orchards of the neighbouring provinces and regions, becoming primarily endemic to areas where the most widespread cultivar of lemon, ‘Femminello’, is produced [3]. The pathogen is currently present along the east coast of the Black Sea (Georgia), and in citrus-growing countries of the Mediterranean basin, except Morocco and Portugal [4], where it is considered as the major destructive fungal disease, with serious economic impact on the Mediterranean citrus industry. The main host of the pathogen is lemon [*C. limon* (L.) Burm. f.], although the disease also has a relevant economic impact on cedar (*C. medica* L.), lime (*C. aurantifolia* Christ.), bergamot (*C. bergamia* Risso), chinotto (*C. myrtifolia* Raf.), sour orange (*C. aurantium* L.), rough lemon (*C. jambiri* Lush), volkamerian lemon (*C. volkameriana* Ten. et Pasq.), and alemow (*C. macrophilla* Wester) [5]. The pathogen enters through wounds, reaches the xylem, and then spreads systemically, causing a range of symptoms that differ according to the site of infection. The most common form of mal secco usually appears in spring, and causes leaf vein and shoot chlorosis, followed by defoliation, wilt and dieback of twigs and branches, and finally the death of the plant [5,6]. The damages caused by the disease can be distinguished directly, i.e., as low yield related to the reduction of canopy volume or tree death, and indirectly, i.e., linked to the higher costs of disease control and the replacement of dead trees in the orchard [6]. Disease management is based on the adoption of preventive measures, including (a) the use of healthy plants from certified nurseries and the use of tolerant cultivars; (b) the disinfection of wounds caused by adverse climatic events (hailstorm, frost, and wind) and human activities (harvesting, pruning, and soil tillage), using copper compounds when climatic conditions are suitable for infection; (c) the use of windbreaks and hail nets that reduce the risk of infection. In the case of infected plants, the disease must be controlled by the costly practice of pruning diseased twigs, which are burned to reduce the inoculum, or by the eradication of the entire plant when it is severely infected [7,8]. Many efforts have already been made regarding the genetic improvement of lemon to enhance its tolerance to mal secco; however, this goal has not yet been achieved due to the absence of a tolerant lemon cultivar with satisfactory productive characteristics [9,10]. For this purpose, several researchers still aim to develop biotechnological approaches, based on molecular markers, to detect mal secco tolerant varieties with optimal fruit quality [10]. Besides cultural practices, disease management depends almost completely on the use of copper compounds. Currently, the use of copper in plant protection is restricted to a maximum application rate of 28 kg ha^−1^ of copper over a period of 7 years [11] in order to minimize the accumulation in soil [12,13], the development of copper-resistant bacterial strains [14,15], and the exposure of non-target organisms, such as insects, beneficial microorganisms in the soil [16], and aquatic organisms [17]. According to the European Commission’s ‘’Farm to Fork Strategy’’, it is necessary to reduce the use of pesticides by 50% by the year 2030, and to give priority to eco-friendly, healthier, and safer alternatives. Many researchers have focused on the use of biological control agents (BCAs) to replace or reduce the use of copper [18,19]. Nevertheless, few studies have been carried out that evaluate the potential activity against *P. tracheiphilus* of endophytic microorganisms colonizing the same ecological niche as the pathogen [20,21]. Moreover, although many BCA formulations are available to the worldwide market to control pre- and post-harvest fungal diseases, none of these have been evaluated against mal secco. Therefore, the aim of this study was to investigate the potential activity of four commercial products, based on *Bacillus amyloliquefaciens* strains, against *P. tracheiphilus* through in vitro and in vivo experiments.

## 2. Results

### 2.1. Isolation and Characterization of P. tracheiphilus Isolates

Flat colonies with sparse hyaline aerial mycelia, and that turned brown or red after a few days, were frequently isolated from symptomatic woody tissues. A total of eight single-spore isolates were collected and molecularly characterized. The comparison of the sequences obtained with those present in NCBI nucleotide database indicated a high identity value (100%) with *P. tracheiphilus* (GenBank Acc. No. MK461058).

### 2.2. In Vitro Activity Evaluation of BCAs against P. tracheiphilus

**Figure 1:**
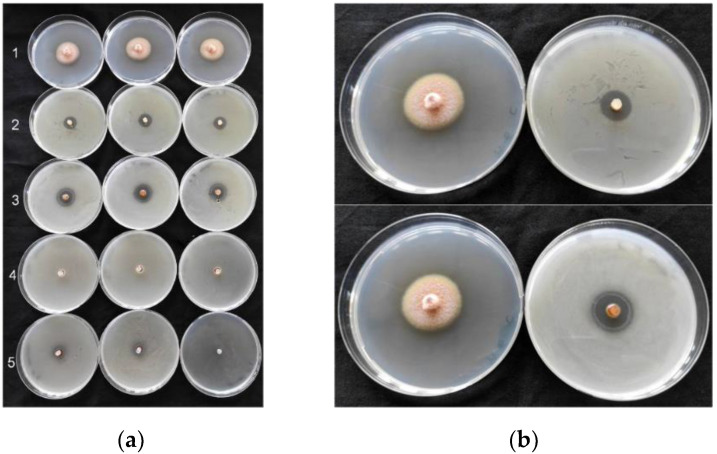
(**a**) Activity of bacterial cells against *Plenodomus tracheiphilus* isolate PT6: 1, control plates; 2, *Bacillus amyloliquefaciens* D747 (Amylo-X^®^); 3, *B. amyloliquefaciens* FZB24 (Taegro^®^); 4, *B. amyloliquefaciens* MBI600 (Serifel^®^); 5, *B. amyloliquefaciens* QST713 (former *B. subtilis*; Serenade^®^Aso). (**b**) Detail of inhibition halos around pathogen colonies induced by *B. amyloliquefaciens* QST713 (former *B. subtilis*; Serenade^®^Aso) (up) and *B. amyloliquefaciens* D747 (Amylo-X^®^)(down).

**Figure 2:**
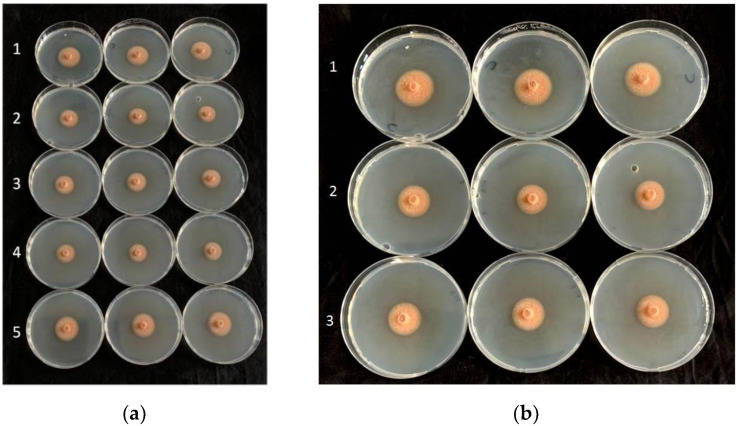
(**a**) Activity of bacterial filtrates against *Plenodomus tracheiphilus* isolate PT6: 1, control plates; 2, *Bacillus amyloliquefaciens* QST713 (former *B. subtilis*; Serenade^®^Aso); 3, *B. amyloliquefaciens* D747 (Amylo-X^®^); 4, *B. amyloliquefaciens* MBI600 (Serifel^®^); 5, *B. amyloliquefaciens* FZB24 (Taegro^®^). (**b**) Detail of bacterial filtrate activity against pathogen: 1, control plates; 2, *B. amyloliquefaciens* QST713 (former *B. subtilis*; Serenade^®^Aso); 3, *B. amyloliquefaciens* D747 (Amylo-X^®^).

**Figure 3:**
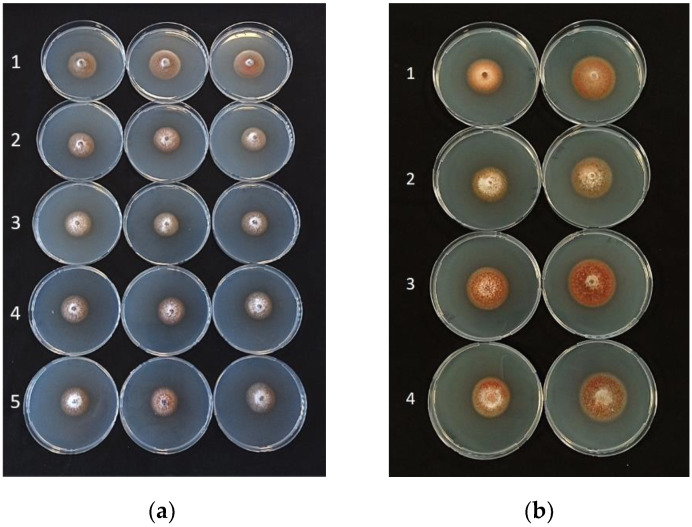
(**a**) Activity of bacterial VOCs against *Plenodomus tracheiphilus* isolate PT5: 1, control plates; 2, *Bacillus amyloliquefaciens* QST713 (former *B. subtilis*; Serenade^®^Aso); 3, *B. amyloliquefaciens* D747 (Amylo-X^®^); 4, *B. amyloliquefaciens* FZB24 (Taegro^®^); 5, *B. amyloliquefaciens* MBI600 (Serifel^®^). (**b**) Effects induced on four different isolates (1–4) of *P*. *tracheiphilus* by VOCs produced by *B. amyloliquefaciens* QST713 (former *B. subtilis*; Serenade^®^Aso) (left) compared with control (right).

The in vitro assays showed consistent, significant effects of the cells, filtrates, and VOCs of the antagonistic bacteria in reducing the mycelial diameters of all isolates of *P*. *tracheiphilus* (Table 1, Table 2 and Table 3; Figure 1, Figure 2 and Figure 3). According to the data shown in these tables, all bacterial cells, filtrates, and VOCs were able to significantly minimize the average mycelial diameter, except for filtrates of *B*. *amyloliquefaciens* strains D747 (Amylo-X^®^) and FZB24 (Taegro^®^) against *P*. *tracheiphilus* isolate PT6. When the antagonistic bacteria were incorporated into the medium as cells, they were more effective at reducing average mycelial growth than the filtrates and VOCs, with average of inhibition percentages ranging from approximately 60 to 96% (Table 1). On the basis of all data for the tested *P*. *tracheiphilus* isolates, *B*. *amyloliquefaciens* strain MBI600 had the strongest effects in terms of reducing fungal mycelial growth, as cells, filtrates, and VOCs, with the exception of its effects as VOCs against *P*. *tracheiphilus* isolate PT3. Strong performances were also detected for the cells, filtrates, and VOCs of *B*. *amyloliquefaciens* QST713 (former *B*. *subtilis*; Serenade^®^Aso), which often induced similar percentage reductions to those detected for *B*. *amyloliquefaciens* MBI600 (Table 1, Table 2 and Table 3).

### 2.3. Virulence Assessment of P. tracheiphilus Isolates

**Figure 4:**
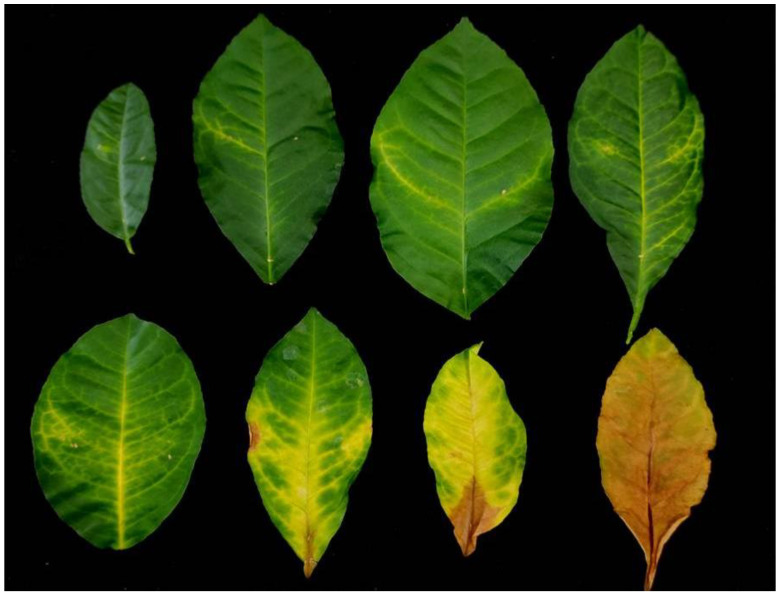
Mal secco disease progression caused by *Plenodomus tracheiphilus* over time on *Citrus volkameriana* leaves.

Regarding the virulence assessment of *P*. *tracheiphilus*, a wide variability was detected among the tested isolates over time (Table 4). The progression of symptoms following the pathogen inoculation is reported in Figure 4. Based on average disease incidence (DI) and symptom severity (SS), isolate PT4 showed the highest aggression, in vivo, against *C*. *volkameriana* leaves, followed by PT3, PT5, and PT1; however, the disease parameter values were not always significantly different among them (Table 4). Based on the overall data, PT6 and PT9 were, relatively, the least virulent isolates. For this reason, the most aggressive PT4 isolate was chosen for the following in vivo experiments performed to compare the in vivo performances of bioformulates and fungicides.

### 2.4. In Planta Activity Evaluation of BCAs against P. tracheiphilus in Comparison with Standard Fungicides 

**Figure 5:**
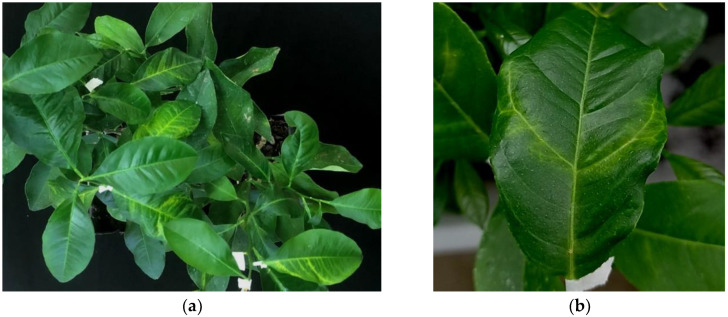
(**a**) Mal secco disease symptoms on artificially inoculated plants; (**b**) detail of leaf veins chlorosis.

In the in vivo experiments on the performances of fungicides and biological active compounds against the fungal pathogen, there was consistently a significant effect of the treatments on the tested parameters, i.e., DI and SS values collected over time (*p* value < 0.0001) (Table 5; Figure 5). Alternatively, the treatment × trial interactions were not significant, thus indicating a similar ranking of effectiveness between the two trials. Consequentially, the two trials were combined (Table 6) and subjected to a post-hoc analysis of the main effects. 

Post-hoc analysis of the treatment effects in reducing infections caused by *P. tracheiphilus* PT4 are reported in Table 6. Based on these data, all fungicides induced the greatest reductions in fungal infections over time on the citrus leaf compared to those detected for antagonistic bacteria (Table 6).

In detail, mancozeb and fludioxonil showed the best performances, since they were able to significantly reduce DI and SS values to a greater degree compared to all the bioformulate applications, except for SS at 20 days (Table 6). This last result could be related to the extremely low disease pressure detected after 20 days, which did not allow to detect significant differences among treatments. Although copper hydroxide consistently exhibited performances against infections similar to those of other fungicides (data not significant), its DI and SS values did not always significantly differ from those recorded for *B.* *amyloliquefaciens* QST713 (former *B. subtilis*) and, only for SS values, also from those detected for *B. amyloliquefaciens* strains MBI600 and FZB24 at 20 and 30 days after pathogen inoculation, respectively. Among all the bio-fungicides tested, only the *B. amyloliquefaciens* strain D747 (Amylo-X) was unable to reduce SS values when limited to 20 days after inoculation (Table 6).

### 2.5. Endophytic Colonization of Woody Stem by Bacillus spp. 

**Figure 6:**
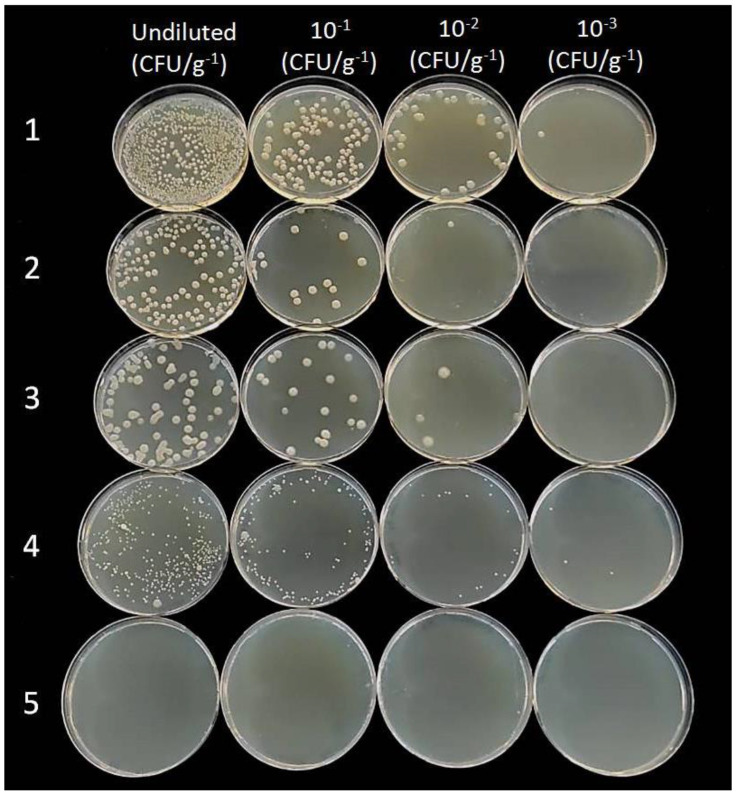
Recovery of bacterial strains from *Citrus volkameriana* woody stems on PDA plates after 48 h compared with control plates: 1, *B. amyloliquefaciens* MBI600 (Serifel^®^); 2, *B. amyloliquefaciens* D747 (Amylo-X^®^); 3, *B*. *amyloliquefaciens* QST713 (former *B. subtilis*; Serenade^®^Aso); 4, *B*. *amyloliquefaciens* FZB24 (Taegro^®^); 5, control plates.

Re-isolation of the BCAs from the internal woody tissue of lemon seedlings, conducted to determine their endophytic capacities, produced positive results showing the growth of all bacterial strains on a nutrient substrate after 48 h. On the contrary, no bacterial colonies were re-isolated from plants used as controls (Figure 6). Bacterial colonies were similar in size, except those isolated from plants treated with *B*. *amyloliquefaciens* strain FZB24 (Taegro^®^), which were smaller than the others. Moreover, they were fast-growing, dull, white, flat, and had slightly irregular margins. The number of cells counted on the culture medium did not differ significantly between treatments, ranging from 10^3^ to 10^4^ CFU/g^−1^. The sequencing of 16S rDNA regions of each *B. amyloliquefaciens* strain, performed by Macrogen Inc., showed 100% homology with species belonging to the group *B*. *amyloliquefaciens* (*B. subtilis* species complex) [22]. 

Although all *Bacillus* strains were recovered from the stems of pre-treated seedlings, demonstrating the ability to survive and achieve endophytic colonization of woody tissues, we consider it necessary that future experiments are carried out to evaluate whether the bacterial strains also move through the vascular system of citrus plants. 

## 3. Discussion

In this study, four biological products based on *B*. *amyloliquefaciens* strains were selected and tested for their antagonistic activity against eight isolates of *P. tracheiphilus*, causal agent of citrus mal secco disease. This disease is the main cause of production losses in the lemon industry of the Mediterranean basin, and also represents a threat for the major lemon producing countries worldwide, where the risk of the disease being introduced through plant material is high. Considering that the current strategies for disease control often prove to be inefficient, and that copper use is continuously subjected to increasingly severe European restrictions, new effective alternatives should be developed to implement integrated control strategies. A sustainable alternative to reduce and/or replace the use of fungicides could be the application of BCAs. Previous studies have shown the antagonistic effects of endophytic bacteria (*B. amyloliquefaciens*, *B*. *subtilis*, and *Pseudomonas fluorescens*), isolated from citrus, against *P. tracheiphilus* [20,21]. Although natural endophytic bacteria could be a potential strategy for the biological control of mal secco disease, careful evaluation of BCAs is needed before their application in the field. Screening procedures prior to commercialization should be carried out to avoid the risk of introducing strains that could be pathogenic to plants or unsafe for humans. For this reason, an easier and safer way is to evaluate the effectiveness of biological products based on *B*. *amyloliquefaciens* strains available in the market. The results obtained from our in vitro experiments showed that all bacterial strains, mainly *B*. *amyloliquefaciens* strain MBI60 (Serifel^®^), were effective in reducing mycelial growth in most of the tested *P. tracheiphilus* isolates. Specifically, the strong activity exhibited by the bacterial cells when incorporated into media is probably related to different modes of action, including the synthesis of antimicrobial substances (diffusible toxic metabolites and volatile organic compounds) and competition for nutrients and space. *Bacillus* spp. are well-known to produce a variety of substances, such as non-ribosomally synthesized peptides and lipopeptides, polyketide compounds, bacteriocins, and siderophores [23,24], which have an inhibitory effect on important fungal pathogens of citrus plants and other crops [25,26,27]. 

The in vivo experiments showed that fludioxonil and mancozeb fungicides exhibited the highest performance in terms of managing disease. Unfortunately, mancozeb was banned in EU states in 2021, and, in the same year, the use of fludioxonyl (Geoxe) was authorized, derogating from the regular process, for a period not exceeding 120 days, only against emerging diseases caused by *Alternaria* and *Colletotrichum* species [28,29,30]. 

These initial data on the effectiveness of fludioxonil against *P*. *tracheiphilus* could be used to develop an alternative strategy to copper treatments. 

Moreover, all biological products showed high biocontrol activity against infections caused by *P. tracheiphilus* in terms of reducing values of DI and SS in *C. volkameriana* seedlings, confirming the results reported for *Bacillus* species in other studies on mal secco disease [20,21]. However, these are the first data outlining the effectiveness of commercial biological products based on *Bacillus* strains against *P*. *tracheiphilus*. Furthermore, *Bacillus* species are considered to be plant growth promoting bacteria and systemic resistance inducers (ISR) [23,31]; therefore, the potential introduction of these action mechanisms promoting crop growth could be an additional benefit of BCAs.

Although further studies should be performed to evaluate these commercial products under field conditions, the high effectiveness of these formulates as preventative treatments against *P. tracheiphilus*, together with their ability to survive and colonize citrus internal tissues, make them suitable for application. Mal secco disease management is based on the adoption of combined preventive measures, including cultural practices and treatments for the disinfection of wounds after pruning or adverse climatic events, and the use of copper compounds. Consequently, the herein tested commercial biological products could be included as effective alternatives to chemical treatments in an integrated strategy for the management of mal secco disease. 

## 4. Materials and Methods

### 4.1. Isolation and Characterization of P. tracheiphilus Isolates

Twig samples were collected from lemon trees with typical salmon-pink wood discoloration, growing in commercial orchards in Sicily. Symptomatic tissue fragments were disinfected with NaOCl (1%), rinsed in sterile distilled water (SDW), and placed onto potato dextrose agar (PDA, Lickson, Vicari, Italy) amended with 100 mg/L with streptomycin sulphate (Sigma-Aldrich, St. Louis, MO, USA) to prevent bacterial growth. Petri plates were incubated at 24 ± 1 °C under dark conditions for 7 days. Identification of isolates was conducted based on morphological characteristics in pure culture. Single conidia were selected from resulting colonies and transferred into PDA plates to obtain single-spore isolates. 

Genomic DNA of eight isolates (named PT1, PT3, PT4, PT5, PT6, PT7, PT8, and PT9) grown on malt extract agar (MEA) was extracted using a DNA extraction Kit (Promega Corporation, Madison, WI, USA), according to the manufacturer’s protocol. The internal transcribed spacer (ITS) of ribosomal DNA region (rDNA) was targeted for PCR amplification using ITS5 and ITS4 primers. The PCR conditions were: initial denaturation at 94 °C for 30 s; 35 cycles of amplification at 94 °C for 30 s; annealing at 50 °C for 1 min; extension at 68 °C for 1 min, followed by a final extension period at 68 °C for 5 min. Following PCR amplification, the amplicons were visualized on a 1% agarose gel stained with GelRed and viewed under ultraviolet light. Amplicons were purified and sequenced in both directions by Macrogen Inc. (Seoul, Korea), and the new sequences obtained were analyzed and aligned manually using MEGAX (molecular evolutionary genetics analysis). The sequences obtained were compared with those present in the NCBI nucleotide database to verify identity (%). 

### 4.2. In Vitro Activity Evaluation of BCAs against P. tracheiphilus

Several in vitro experiments were carried out to evaluate the activity of bacterial cells, volatile organic compounds (VOCs), and cell-free culture filtrates of *B*. *amyloliquefaciens* QST713 (former *B*. *subtilis*; Serenade^®^Aso Aso, Bayer CropScience S.r.l., Milano, Italy), *B*. *amyloliquefaciens* subsp. *plantarum* D747 (Amylo-X^®^ LC, Biogard, Mitsui AgriScience International S.A./N.V., Brussels, Belgium), *B*. *amyloliquefaciens* FZB24 (Taegro^®^, Syngenta, Novozymes A/S., Bagsvaerd, Denmark), and *B*. *amyloliquefaciens* MBI600 (Serifel^®^, BASF, S.P.A., Cesano Maderno, Italy) against *P. tracheiphilus* isolates. To investigate the effectiveness of bacterial strains, experiments were performed using the methods described by Aiello et al. [32], slightly modified.

#### 4.2.1. Activity of BCA Cells

Bacterial colonies were obtained from the microbiological products by streaking a suspension on nutrient agar (NA, Oxoid, Basingstoke Hampshire, UK) at the label rates. After 48 h of incubation at 25 ± 1 °C, bacterial suspension (1 mL), with a concentration of approximately 1 × 10^9^ CFU mL^−1^, was added to Petri plates containing 14 mL of potato dextrose agar (PDA) medium, and maintained at 45 °C. As soon as the PDA was completely solidified, a cylindrical mycelial plug was taken from the edge of *P. tracheiphilus* colony by a sterile cork-borer and placed into the center of the plate. Controls were represented by plates containing PDA and inoculated only with the mycelial plug. After 7 days of incubation at 24 ± 1 °C, the biocontrol activity was evaluated by measuring diameters of the inhibition zone formed between fungal and bacterial colonies. The experiment was replicated three times and performed twice. 

#### 4.2.2. Activity of Cell-Free Culture Filtrates

*Bacillus* strains were grown on nutrient agar (NA) and incubated overnight at 25 °C ± 1 °C. Bacterial suspension was placed into conical flasks containing nutrient broth (NB) and incubated at 25 °C ± 1 °C. After 48 h, the flasks were centrifuged at 9000 rpm for 20 min, and the supernatants were recovered using 0.22 µm filters (LLG Syringe Filter CA, Meckenheim, Germany) to add them to the plates containing PDA. As soon as the PDA was solidified, a mycelial plug of the pathogen was taken from the active growing edge of the colony and placed into the PDA plate. Control was represented by PDA plates containing only the pathogen. After 7 days of incubation at 24 ± 1 °C, the diameter of the fungal mycelium was measured, and the radial growth reduction was calculated as previously described. The experiment was replicated three times and performed twice.

#### 4.2.3. Activity of Volatile Organic Compounds (VOCs)

An aliquot (100 µL) of bacterial suspension, obtained as described above, was streaked onto the PDA plates with a sterilized needle eye and incubated at 25 °C ± 1 °C for 48 h. Subsequently, a mycelial plug of *P. tracheiphilus* was taken from the active growing edge of the colony and placed into another PDA plate. The plates inoculated with the antagonists and the pathogen were then covered, sealed with Parafilm to prevent loss of volatile substances, and incubated at 24 °C ± 1 °C. Controls were represented by PDA plates containing only the pathogen. After 7 days, the diameter of the fungal colony was measured and the biocontrol activity was evaluated by calculating the percentage inhibition of mycelial growth (PGI), using the following formula:

$$\mathrm{PGI}=\frac{\mathrm{Dc}-\mathrm{Da}}{\mathrm{Dc}}\times 100$$

where: PGI is the percentage inhibition of mycelial growth; Dc is the diameter of the fungal colony in the control plate; and Da is the diameter of the fungal colony in the presence of the antagonist. The experiment was replicated three times and performed twice. 

### 4.3. Virulence Assessment of P. tracheiphilus Isolates

Eleven-month-old seedlings (*C*. *volkameriana*) were used to evaluate differences between eight isolates of *P. tracheiphilus* in terms of virulence. Six seedlings were used in this experiment and three apical leaves per seedling were chosen to be inoculated in three different midveins. Therefore, a total of 54 inoculation points per treatment were conducted. Apical leaves of healthy condition were wounded at the midveins with a sterile needle, and a drop of 20 μL of conidial suspension (1 × 10^5^ CFU/mL), obtained from 20-day-old colonies of *P. tracheiphilus* grown on PDA, was placed on each wound using a Gilson micropipette. Seedlings were transferred to the growth chamber at 25°C and 80% of UR. Thirty days after pathogen inoculation, DI and SS parameters were evaluated. The DI value was referred to as the assessment of the percentage of positive inoculation points, whereas the SS value was counted on each inoculation point, adopting the empirical 0-to-4 rating scale of Luisi et al. [33], where: 0, no symptom; 1, chlorotic halo around the inoculation point; 2, chlorosis of the vein close to the inoculation point; 3, extended vein chlorosis to the leaf margin; 4, extensive vein chlorosis and/or browning close to the inoculation point. Symptom severity was calculated using the following formula:

$$\mathrm{SS}=\frac{\sum_{i=0}^{n}\left(\mathrm{Ci}\;\times \;n\right)}{N}$$

where: SS is the average index of severity symptoms; Ci is each class detected; n is the number of inoculation points in each class; i (0-to-4) are the numerical values of the classes; N is the total number of inoculation points examined. Each treatment was replicated three times and the experiment was performed twice. 

### 4.4. In Planta Activity Evaluation of BCAs against P. tracheiphilus 

A total of 7 commercial products were tested in this experiment using 192 eleven-month-old citrus seedlings (*C. volkameriana*) grown under growth chamber conditions, with 24 plants per treatment. The same number of seedlings inoculated only with the pathogen was used as a control. Products tested were microbiological formulates used in the previous in vitro experiments and three standard fungicides used on citrus fruits until recently (products, characteristics, and rates used are reported in the Table 7). BCAs were applied 7 days and 1–2 h before the pathogen inoculation, whereas the fungicides were only applied once, 1–2 h before the pathogen inoculation. Four leaves per seedling were wounded at two midveins with a sterile needle before the final BCA treatment and fungicide application. The treatments were performed by spraying a volume of 100–150 mL of suspension onto the leaves, and the pathogen was inoculated onto each wounded leaf by spraying approximately 0.8 mL of conidial suspension (1 × 10^5^ CFU/mL), obtained from 20-day-old colonies of PT4. Seedlings were transferred to the growth chamber at 25 °C and 80% of UR. Twenty and 30 days after the pathogen inoculation, DI and SS parameters were evaluated as previously described. Each treatment was replicated three times and the experiment was performed two times. 

### 4.5. Endophytic Colonization of Woody Stem by Bacillus spp.

Eleven-month-old citrus seedlings (*C. volkameriana*) were used to assess the endophytic colonization ability of *Bacillus* strains used in the previous experiments. Each BCA was applied to three seedlings two times, 7 days apart. Treatments were performed by spraying 100–150 mL of bacterial suspension onto the leaves. Seedlings were transferred to the growth chamber at 25 °C and 80% of UR. After 50 days, plants were cut into segments at regular intervals along the stem, and tissues were disinfected using the methods described by Araújo et al. [34], slightly modified. Stem fragments were rinsed in 70% ethanol for 5 min, surface-disinfected with 2% sodium hypochlorite solution for 5 min, rinsed once in 70% ethanol for 30 s and twice in sterile distilled water. In order to check the success of the disinfection process, aliquots of the water used for the final rinse were streaked onto NA. Then, the bark of surface-disinfected stem was removed, and the inner part was homogenized in 10 mL of sterile phosphate-buffered saline (containing NaCl at 8 g/L, KCl at 0.2 g/L, Na2HPO4 at 1.4 g/L, and KH2PO4 at 0.24 g/L) using a sterile blade to set up the dilution series. An aliquot (0.1 mL) was taken from each dilution, placed into the PDA plates, and spread using a sterile hockey stick. After incubation at 28 °C, bacterial cells were identified using morphological characteristics and hand-counted to estimate population density. Moreover, the genomic DNA of bacterial colonies was extracted and the 16S genes of ribosomal DNA region (rDNA) was targeted for PCR amplification by Macrogen Inc. The sequences obtained were analyzed, aligned manually using MEGAX, and compared with those present in the NCBI nucleotide database. 

### 4.6. Data Analysis

All statistical analyses of in vitro and in vivo data were performed by using the Statistica software package (version 10; Statsoft Inc., Tulsa, OK, USA). The arithmetic means of mycelial diameter, DI, and SS data were calculated by averaging the values determined for the single replicates of each treatment, and reported in the tables. Percent DI data were arcsine (sin^−1^ square root x) transformed to meet assumptions of homogeneity of variance. First analyses of DI and SS were performed by calculating values for F and the associated p values to evaluate whether the effects of a single factor, and treatment × trial interactions, were significant. In the post-hoc analyses, main effects of treatments were evaluated with an analysis of variance, and the means were separated by the Fisher’s least significant difference test (α = 0.05).

## Figures and Tables

**Table 1:** In vitro effects of selected antagonistic bacterial cells in terms of reducing mycelial diameter in eight representative *Plenodomus tracheiphilus* isolates.

Treatment	Average Mycelial Growth (cm) of *Plenodomus tracheiphilus* Isolates (PT) ^1^							
	PT1	PT3	PT4	PT5	PT6	PT7	PT8	PT9
Control	3.27 ± 0.01 a	2.68 ± 0.06 a	2.73 ± 0.04 a	3.37± 0.01 a	2.95 ± 0.07 a	2.73 ± 0.11 a	3.17 ± 0.05 a	3.38 ± 0.15 a
*B. amyloliquefaciens* D747 (Amylo-X^®^)	1.03 ± 0.03 b	1.05 ± 0.03 b	0.95 ± 0.03 b	1.07 ± 0.06 b	0.90 ± 0.05 b	0.65 ± 0.12 b	0.92 ± 0.05 b	1.00 ± 0.11 b
*B. amyloliquefaciens* FZB24 (Taegro^®^)	0.48 ± 0.08 c	0.78 ± 0.05 c	0.63 ± 0.03 c	0.40 ± 0.04 c	0.42 ± 0.13 c	0.45 ± 0.07 bc	0.50 ± 0.00 c	0.42 ± 0.04 c
*B. amyloliquefaciens* MBI600 (Serifel^®^)	0.52 ± 0.05 c	0.62 ± 0.08 c	0.53 ± 0.04 c	0.48 ± 0.06 c	0.30 ± 0.05 cd	0.43 ± 0.03 bc	0.28 ± 0.01 d	0.37 ± 0.03 c
*B. amyloliquefaciens* QST713 (former *B. subtilis*; Serenade^®^Aso)	0.23 ± 0.03 d	0.38 ± 0.04 d	0.18 ± 0.08 d	0.20 ± 0.00 d	0.10 ± 0.05 d	0.18 ± 0.08 c	0.20 ± 0.00 d	0.33 ± 0.08 c

^1^ Data from three replicates (PDA plates) with standard error of the mean (± SEM). Values followed by different letters within the column are significantly different according to Fisher’s least significance differences test (α = 0.05).

**Table 2:** In vitro effects of selected antagonistic bacterial filtrates in terms of reducing mycelial diameter in eight representative *Plenodomus tracheiphilus* isolates.

Treatment	Average Mycelial Growth (cm) of *Plenodomus tracheiphilus* Isolates ^1^							
	PT1	PT3	PT4	PT5	PT6	PT7	PT8	PT9
Control	2.48 ± 0.01 a	2.57 ± 0.04 a	2.68 ± 0.08 a	2.48 ± 0.06 a	2.77 ± 0.23 a	2.58 ± 0.07 a	3.25 ± 0.04 a	2.60 ± 0.05 a
*B. amyloliquefaciens* D747 (Amylo-X^®^)	2.27 ± 0.03 b	2.33 ± 0.03 b	2.25 ± 0.07 b	1.87 ± 0.11 b	2.50 ± 0.03 abc	1.70 ± 0.16 c	2.45 ± 0.03 bc	2.20 ± 0.07 b
*B. amyloliquefaciens* FZB24 (Taegro^®^)	2.27 ± 0.08 b	2.23 ± 0.06 bc	2.20 ± 0.10 b	1.98 ± 0.01 b	2.73 ± 0.03 ab	2.25 ± 0.07 ab	2.58 ± 0.04 b	2.07 ± 0.12 b
*B. amyloliquefaciens* MBI600 (Serifel^®^)	1.97 ± 0.01 c	2.13 ± 0.10 cd	1.47 ± 0.01 c	1.55 ± 0.00 c	2.35 ± 0.08 bc	1.58 ± 0.21 c	2.47 ± 0.18 bc	1.72 ± 0.01 c
*B. amyloliquefaciens* QST713 (former *B. subtilis*; Serenade^®^Aso)	1.87 ± 0.07 c	1.98 ± 0.01 d	1.68 ± 0.10 c	1.62 ± 0.03 c	2.23 ± 0.01 c	1.87 ± 0.18 bc	2.02 ± 0.26 c	1.63 ± 0.04 c

^1^ Data from three replicates (PDA plates) with standard error of the mean (± SEM). Values followed by different letters within the column are significantly different according to Fisher’s least significance differences test (α = 0.05).

**Table 3:** In vitro effects of selected antagonistic bacterial VOCs in terms of reducing mycelial diameter in eight representative *Plenodomus tracheiphilus* isolates.

Treatment	Average Mycelial Growth (cm) of *Plenodomus tracheiphilus* Isolates ^1^							
	PT1	PT3	PT4	PT5	PT6	PT7	PT8	PT9
Control	3.22 ± 0.11 a	2.97 ± 0.02 a	3.20 ± 0.00 a	3.18 ± 0.06 a	3.33 ± 0.04 a	3.02 ± 0.06 a	3.37 ± 0.03 a	3.28 ± 0.03 a
*B. amyloliquefaciens* D747 (Amylo-X^®^)	2.78 ± 0.04 b	2.80 ± 0.03 b	2.67 ± 0.04 b	2.58 ± 0.04 b	2.33 ± 0.07 c	2.30 ± 0.06 b	2.57 ± 0.09 c	2.68 ± 0.06 b
*B. amyloliquefaciens* FZB24 (Taegro^®)^	2.70 ± 0.08 bc	2.65 ± 0.03 c	2.50 ± 0.05 b	2.72 ± 0.02 b	2.45 ± 0.08 bc	2.32 ± 0.12 b	2.47 ± 0.09 c	2.43 ± 0.07 c
*B. amyloliquefaciens* MBI600 (Serifel^®^)	2.42 ± 0.20 c	2.67 ± 0.03 c	2.50 ± 0.1 b	2.62 ± 0.06 b	2.62 ± 0.04 b	2.52 ± 0.04 b	2.92 ± 0.13 b	2.65 ± 0.08 b
*B. amyloliquefaciens* QST713 (former *B. subtilis*; Serenade^®^Aso)	2.37 ± 0.07 c	2.78 ± 0.04 b	2.60 ± 0.15 b	2.68 ± 0.03 b	2.52 ± 0.04 b	2.52 ± 0.21 b	2.60 ± 0.08 c	2.57 ± 0.03 bc

^1^ Data from three replicates (PDA plates) with standard error of the mean (± SEM). Values followed by different letters within the column are significantly different according to Fisher’s least significance differences test (α = 0.05).

**Table 4:** Compared virulence of eight *Plenodomus tracheiphilus* isolates over time.

*P. tracheiphilus* Isolates	DI (%) 10 dai ^1^	SS 10 dai ^1^	DI (%) 20 dai ^1^	SS 20 dai ^1^	DI (%) 30 dai ^1^	SS 30 dai ^1^
PT1	37.0 ± 6.7 d	0.8 ± 0.1 c	46.3 ± 5.0 cd	1.1 ± 0.2 b	48.0 ± 4.0 cd	1.4 ± 0.2 c
PT3	42.7 ± 10.5 cd	1.0 ± 0.3 bc	42.7 ± 10.5 d	1.2 ± 0.4 b	42.7 ± 10.5 d	1.4 ± 0.3 c
PT4	46.3 ± 3.7 bcd	1.2 ± 0.2 abc	52.0 ± 4.0 bcd	1.6 ± 0.2 ab	54.0 ± 2.0 bcd	2.2 ± 0.3 bc
PT5	48.0 ± 6.7 bcd	1.3 ± 0.1 abc	66.7 ± 3.2 ab	2.4 ± 0.1 a	72.3 ± 3.2 ab	2.8 ± 0.1 ab
PT6	57.3 ± 6.9 abcd	1.5 ± 0.1 ab	68.3 ± 3.7 ab	2.1 ± 0.1 a	68.3 ± 3.7 abc	2.5 ± 0.2 ab
PT7	65.0 ± 6.7 ab	1.2 ± 0.2 abc	68.7 ± 9.3 ab	2.3 ± 0.5 a	66.7 ± 11.3 abc	2.4 ± 0.5 ab
PT8	63.3 ± 3.7 abc	1.6 ± 0.3 ab	65.0 ± 4.7 abc	1.8 ± 0.3 ab	68.7 ± 7.8 abc	2.7 ± 0.3 ab
PT9	72.33 ± 8.3 a	1.74 ± 0.1 a	79.7 ± 5.0 a	2.3 ± 0.1 a	83.3 ± 5.7 a	3.1 ± 0.3 a

^1^ Data derived from three replicates, each formed from 18 leaves belonging to young *Citrus volkameriana* seedlings. SEM = standard error of the mean. Means followed by different letters within the column are significantly different according to Fisher’s least significance differences test (α = 0.05). DI = disease incidence; SS = symptom severity; dai = days after inoculation.

**Table 5:** Analysis of variance for disease incidence and severity over time (after 20 and 30 days) among eight different biological and chemical treatments in two trials.

Factor(s)	df	Parameters ^1^							
		DI (%) 20 dai		SS (1-to-5) 20 dai		DI (%) 30 dai		SS (1-to-5) 30 dai	
		F	*p* Value	F	*p* Value	F	*p* Value	F	*p* Value
*Treatment*	7	94.424	0.000003	35.322	0.006373	12.356	0.000000	84.939	0.000007
*Treatment × trial*	7	14.588	0.2172 ^ns^	0.8841	0.5300 ^ns^	0.8650	0.5440 ^ns^	0.9814	0.4617 ^ns^

^1^ F test for fixed effects and *p* values associated with F; df = degrees of freedom; ns = not significant.

**Table 6:** Post-hoc analysis on main effects of treatments in reducing artificial infections caused by *Plenodomus tracheiphilus* PT4.

Treatment	DI (%) 20 dai ^1^	SS 20 dai ^1^	DI (%) 30 dai ^1^	SS 30 dai ^1^
Control	39.17 ± 0.50 a	0.72 ± 0.08 a	43.33 ± 5.66 a	1.07 ± 0.19 a
*B. amyloliquefaciens* D747(Amylo-X^®^)	28.33 ± 7.33 b	0.55 ± 0.32 ab	30.50 ± 12.96 b	0.73 ± 0.57 b
*B. amyloliquefaciens* MBI600 (Serifel^®^)	25.00 ± 6.63 b	0.43 ± 0.17 bc	27.83 ± 12.02 b	0.63 ± 0.38 bc
*B. amyloliquefaciens* FZB24 (Taegro^®^)	24.50 ± 8.83 b	0.45 ± 0.18 bc	28.17 ± 16.26 b	0.63 ± 0.33 bc
*B. amyloliquefaciens* QST713 (former *B. subtilis*; Serenade^®^Aso)	23.00 ± 6.67 bc	0.40 ± 0.20 bc	26.17 ± 12.02 bc	0.58 ± 0.40 bc
Copper hydroxide (Kocide 2000^®^)	16.00 ± 2.67 cd	0.30 ± 0.17 c	18.83 ± 7.31 cd	0.42 ± 0.31 cd
Fludioxonil (Geoxe^®^)	15.33 ± 9.00 d	0.33 ± 0.23 c	16.17 ± 13.44 d	0.38 ± 0.40 d
Mancozeb (Penncozeb^®^ DG)	14.67 ± 9.67 d	0.30 ± 0.27 c	15.00 ± 13.20 d	0.38 ± 0.45 d

^1^ Data derived from two combined trials, plus/minus SEM (standard error of the mean). Means for each trial derived from three replicates, each formed by 64 inoculation points on 32 leaves belonging to young *Citrus volkameriana* seedlings. Values followed by different letters within the column are significantly different according to Fisher’s least significance differences test (α = 0.05). DI = disease incidence; SS = symptom severity; dai = days after inoculation.

**Table 7:** Bioformulates and fungicides selected for in planta experiments.

Active Ingredient	Trade Name	Manufacturer	Rates (g or mL/100 L)	Formulation ^1^
*Bacillus amyloliquefaciens* strain QST713 (former *B*. *subtilis*)	Serenade^®^Aso Aso	Bayer Crop Science S.r.l., Milano, Italy	600	1.34 SC
*Bacillus amyloliquefaciens* subsp. *plantarum* strain D747	Amylo-X^®^	Biogard, Brussels, Belgium	350	5 LC
*Bacillus amyloliquefaciens* strain FZB24	Taegro^®^	Syngenta, Bagsvaerd, Denmark	25	13 WP
*Bacillus amyloliquefaciens* strain MBI600	Serifel^®^	BASF S.p.A. Italia, Cesano Maderno, Italia	50	8.8 WP
Copper hydroxide	Kocide 2000^®^	Certis Europe B.V., Saronno (VA), Italy	150	35 WG
Mancozeb	Penncozeb^®^ DG	UPL Italia S.r.l., S. Carlo di Cesena (FC) Italy	300	75 WG
Fludioxonil	Geoxe^®^	Syngenta Italia S.p.A., Milano, Italy	40	50 WG

^1^ Percentage of active ingredient. SC, suspension concentrate; LC, liquid formulation; WG, water dispersible granule; WP, wettable powder.

## Data Availability

The data presented in this study are available on request from the corresponding author.
